# Lipid rafts control P2X3 receptor distribution and function in trigeminal sensory neurons of a transgenic migraine mouse model

**DOI:** 10.1186/1744-8069-7-77

**Published:** 2011-09-29

**Authors:** Aswini Gnanasekaran, Mayya Sundukova, Arn MJM van den Maagdenberg, Elsa Fabbretti, Andrea Nistri

**Affiliations:** 1 Neurobiology Sector and Italian Institute of Technology Unit, International School for Advanced Studies (SISSA), Via Bonomea 265, 34136 Trieste, Italy; 2 Department of Neurology, Leiden University Medical Centre, 2300 RC Leiden, The Netherlands; 3 Department of Human Genetics, Leiden University Medical Centre, 2300 RC, Leiden, The Netherlands; 4 Laboratory for Environmental Research, University of Nova Gorica, Vipavska 13, PO Box 301, Rožna Dolina, SI-5000, Slovenia

**Keywords:** neuronal sensitisation, purinergic signalling, membrane domains, ATP

## Abstract

**Background:**

A genetic knock-in mouse model expressing the R192Q mutation of the α1-subunit of the Ca_V_2.1 channels frequently found in patients with familial hemiplegic migraine shows functional upregulation of ATP-sensitive P2X3 receptors of trigeminal sensory neurons that transduce nociceptive inputs to the brainstem. In an attempt to understand the basic mechanisms linked to the upregulation of P2X3 receptor activity, we investigated the influence of the lipid domain of these trigeminal sensory neurons on receptor compartmentalization and function.

**Results:**

Knock-in neurons were strongly enriched with lipid rafts containing a larger fraction of P2X3 receptors at membrane level. Pretreatment with the Ca_V_2.1 channel blocker ω-agatoxin significantly decreased the lipid raft content of KI membranes. After pharmacologically disrupting the cholesterol component of lipid rafts, P2X3 receptors became confined to non-raft compartments and lost their functional potentiation typically observed in KI neurons with whole-cell patch-clamp recording. Following cholesterol depletion, all P2X3 receptor currents decayed more rapidly and showed delayed recovery indicating that alteration of the lipid raft milieu reduced the effectiveness of P2X3 receptor signalling and changed their desensitization process. Kinetic modeling could reproduce the observed data when slower receptor activation was simulated and entry into desensitization was presumed to be faster.

**Conclusions:**

The more abundant lipid raft compartment of knock-in neurons was enriched in P2X3 receptors that exhibited stronger functional responses. These results suggest that the membrane microenvironment of trigeminal sensory neurons is an important factor in determining sensitization of P2X3 receptors and could contribute to a migraine phenotype by enhancing ATP-mediated responses.

## Background

Over the last 20 years, the view of the cell plasma membrane has evolved from that of a homogeneous arrangement of lipids with embedded proteins towards the notion of a mosaic of microdomains endowed with specific lipid and protein composition [1,2]. This finding led to the definition of lipid rafts [3], namely membrane microdomains whose major structure is made up by sphingolipids, glycosylphosphatidylinositol (GPI)-anchored proteins and cholesterol (insoluble in Triton X-100 and with characteristic density fractionation; [4]). Proteins such as Src family kinases can be found in lipid rafts and represent potential modulators of receptors in this compartment [5,6]. Important properties of lipid rafts are their rapid association and dissociation [7,8], as well as changes in their composition dependent on the environment or the physiological state of the cell [9] or the binding of ligands to associated receptors [10].

Growing evidence indicates that lipid rafts are crucial for many neuronal functions, including maintenance and function of several ionotropic receptors for acetylcholine [11,12], and glutamate [13,14]. Purinergic P2X receptors have also been found in lipid rafts [15-17]. Among them, the P2X3 receptor is almost exclusively expressed by sensory ganglion neurons [18,19], where it exerts an important role as transducer of pain [20]. Under basal conditions, the P2X3 subtype is a fast-desensitizing receptor, but it can produce larger currents under pathological pain states [20,21]. The present study aimed at investigating whether lipid rafts might contribute to the expression and function of P2X3 receptors in sensory ganglia in a transgenic migraine mouse model that may shed light on the mechanisms leading to migraine.

Only a few studies looked into the association of P2X3 receptors with lipid rafts in dorsal root ganglia [15,22,23] or recombinant expression systems such as HEK293 cells [17]. The dynamics of this association and whether it has functional consequences are, however, poorly understood. For example, despite the fact that the lipid raft cholesterol is depleted by treatment with methyl β-cyclodextrin (MβCD; [24]), this protocol apparently leaves P2X3 receptors unaffected [17].

Here we investigated the localization of P2X3 receptors in sensory neurons of trigeminal ganglia (TG) from wild type (WT) mice as well as from a genetic knock-in (KI) mouse model that expresses an R192Q missense mutation in the α1 subunit of Ca_V_2.1 channels that leads to human familial hemiplegic migraine type 1 (FHM-1; [25,26]). We recently reported that trigeminal ganglion neurons of the KI mice possess enhanced functional responses of P2X3 receptors without changes in their protein expression level [27]. Hence, we explored whether P2X3 displayed differential localization between lipid rafts and the non-raft compartments depending on the genetic model, and whether this redistribution might have influenced receptor function measured as patch-clamp currents.

## Results

### Preferential localization of P2X3 receptors to lipid rafts of KI trigeminal ganglion neurons

**Figure 1:**
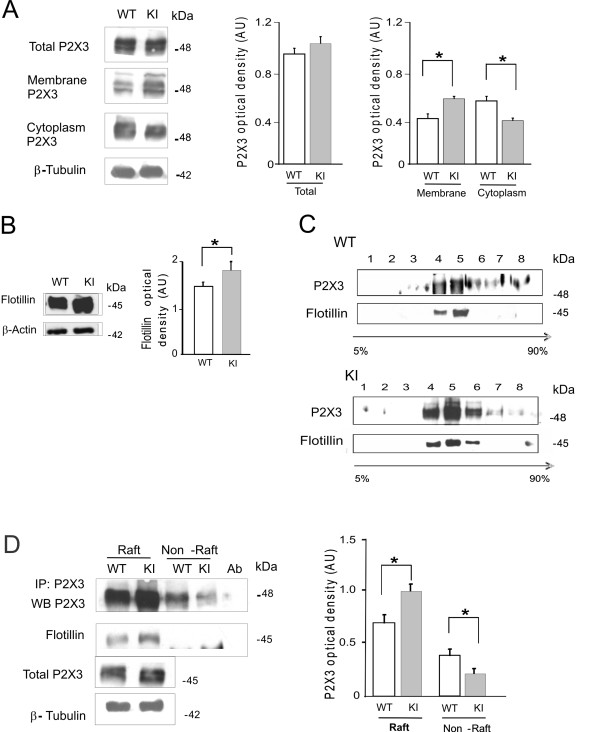
**Expression of P2X3 receptors in the raft domain of trigeminal neurons in situ**. A, left, Examples of western immunoblots of trigeminal ganglion lysates from WT or KI mice labeled with an anti-P2X3 antibody. Gel loading is shown with β-tubulin signal. Middle, Histograms compare expression of P2X3 receptors in total lysates from WT and KI ganglia (n = 5). Right, Histograms quantify stronger expression of P2X3 receptors in membrane compartments of KI samples (n = 5, p = 0.0072). Total P2X3 protein level was normalized with respect to β-tubulin. Membrane or cytoplasmic contents were expressed a fraction of total P2X3 expression. B, left, Examples of western blots (48 kDa) of trigeminal ganglion lysates (from WT or KI mice) immunostained with the anti-flotillin antibody. Equal gel loading is shown with β-actin signal. Right, histograms show significant difference between WT and KI samples (n = 5, p = 0.05). C, western blotting of sucrose density gradient fractions of trigeminal ganglion lysates from WT (top rows) or KI (bottom rows) immunostained with anti-P2X3 and anti-flotillin antibodies. Note discrete localization of P2X3 subunits to flotillin enriched fractions. D, left, Example of western blotting of immunopurified P2X3 receptors from membrane raft and non-raft fractions. Total input for P2X3 receptors is also shown together gel loading input with β-tubulin. Flotillin bands indicate the raft fractions. Right, Histograms quantify significantly larger expression of P2X3 receptors in the raft fraction of KI ganglion neurons (p = 0.008; n = 5). The P2X3 expression at cytoplasm level was significantly lower in KI samples vs WT ones (p = 0.05; n = 5).

In accordance with our previous report [27], we observed comparable expression of P2X3 receptor in WT and KI ganglia in situ (n = 5, p > 0.05; Figure 1A middle panel). Appearance of different gel bands (Figure 1A, left) was accounted for by distinct states of maturation and glycosylation of the receptor subunits with different gel mobility [28,29]. Nonetheless, purification of P2X3 subunits from WT or KI ganglia into membrane (comprising surface and intracellular components) or the lighter cytoplasmic fractions (Figure 1A right) indicated that the level of P2X3 in membranes (calculated with respect to the total lysate) was significantly (p = 0.007) higher for KI than WT samples (Figure 1A, right), while the opposite was observed for the soluble cytoplasmic fraction (Figure 1A, right, p = 0.007). These data suggested distinct subcellular distribution of these receptors according to the molecular phenotype of KI.

We next explored if distinct membrane domains, namely raft and non-raft fractions, were involved in such a differential expression of P2X3 receptors for WT and KI ganglia. Thus, we first investigated the expression of flotillin, a canonical marker of lipid rafts [30], that was per se significantly (p = 0.05) larger in lysates of KI ganglia with respect to WT ones (Figure 1B). Thereafter, using sucrose density gradient experiments and a flotillin antibody (Figure 1C), we could detect more abundant P2X3 receptors in KI raft fractions (bottom rows, lanes 4, 5, 6) than in WT ones (top rows, lanes 5 and 6). Immunoprecipitation of P2X3 receptors from Triton X-100 soluble and insoluble membrane preparations (corresponding to non-raft or raft domains, respectively; Figure 1D, left), confirmed that KI samples expressed more receptors in the raft than in the non-raft fraction (p = 0.008; Figure 1D, right). These data suggested differential distribution of P2X3 receptors at membrane level with a stronger density in membrane lipid rafts of KI ganglia. We, thus, enquired if the larger P2X3 mediated-current amplitude previously found in primary cultures of KI trigeminal neurons [27] might be the result of the preferential receptor segregation to this membrane sub-domain.

**Figure 2:**
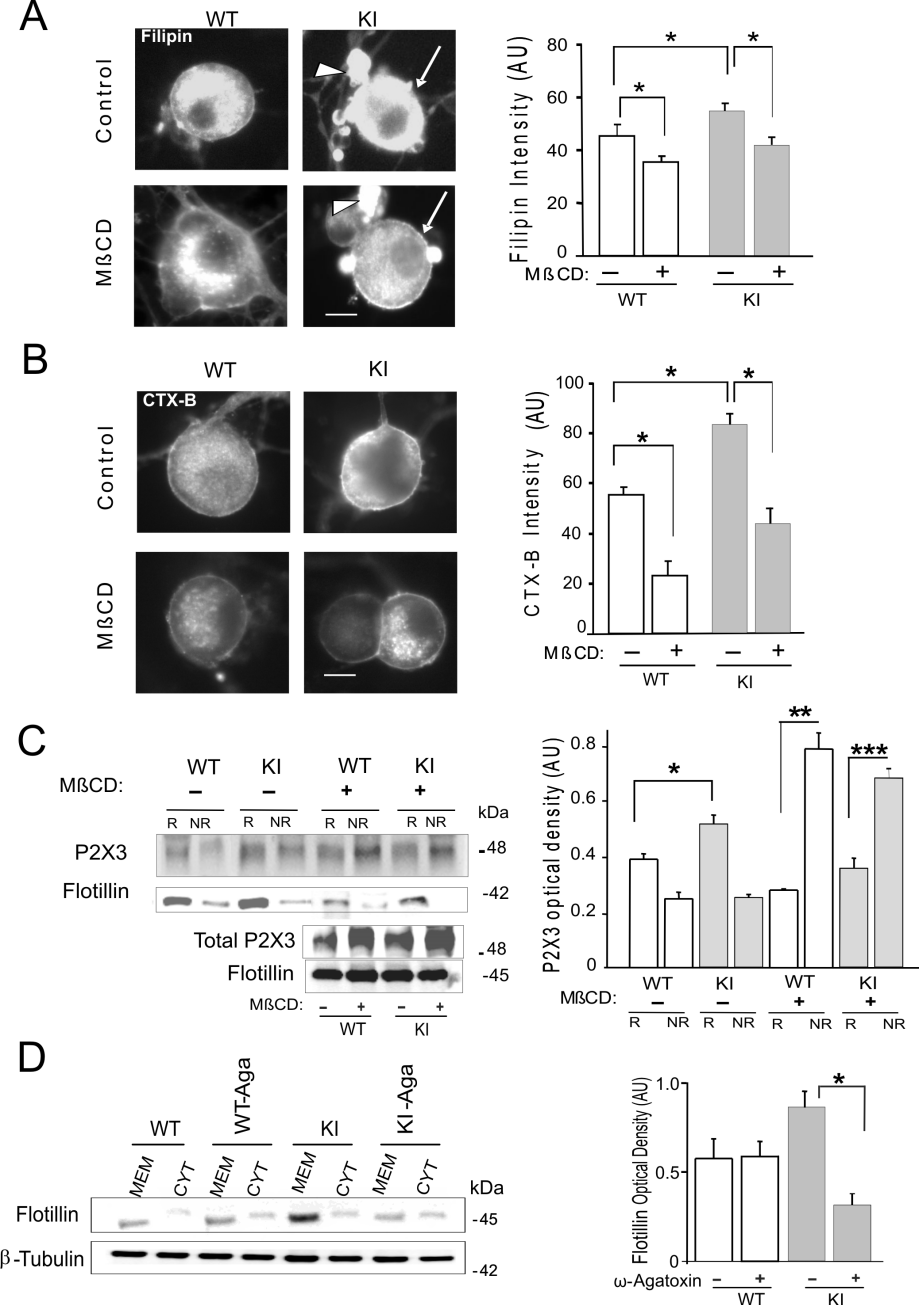
**Effect of MβCD treatment on WT or KI ganglion cultures**. A, Left, cholesterol staining (with filipin) of WT or KI trigeminal neurons (arrow) before and after MβCD (30 min, 10 mM). Satellite cells (arrowhead) are also stained. Bar=10 μm. Right,  fall in cholesterol staining after MβCD (indicated by +) for WT or KI neurons (n= 55); p=0.02 for WT before and after MβCD; p=0.03 for untreated WT vs untreated KI; p=0.01 for KI before and after MβCD. B, CTX-B staining of WT and KI trigeminal neurons before and after MβCD. Bar=10 μm. Right, Large reduction in CTX-B staining after MβCD in WT (p<0.001) and in KI cells (p<0.001). The CTX-B staining of untreated KI cells was significantly larger vs untreated WT neurons (p<0.002); n=62 cells. C, Raft (R) and non rafts (NR) fractions from WT and KI lysates before or after MβCD. Non raft fractions were cytosolic samples collected from supernatants after ultracentrifugation with a swing bucket rotor. Conversely, membrane pellets labelled with flotillin were considered to be raft fractions. Immunolabelling was performed with anti-P2X3 receptor and anti-flotillin antibodies. The histograms (right) quantify the distribution of P2X3 receptors to raft and non- raft compartments of WT or KI cultures: the raft fractions of KI was significantly larger than the corresponding WT ones (*: p=0.004, n=3). After 30 min application of MβCD there was redistribution of P2X3 receptors to cytosolic fractions of WT or KI samples. ** indicates p=0.0038, while *** indicates p=0.001 (n=3). D, example of immunoblotting of P2X3 receptors from supernatants and pellets collected after ultracentrifugation with a fixed angle rotor. The flotillin labelled membrane fractions of KI cultures were largely decreased by overnight pretreatment with ω-agatoxin (300 nM; selective blocker of P/Q channels) as indicated by the histograms (right; * shows p=0.02, n=4).

A standard approach to address this issue was to disrupt lipid rafts by decreasing their membrane cholesterol content with MβCD [17,22] and to find out if the expression and function of P2X3 receptors was subsequently changed. Hence, on cultured trigeminal neurons, labelling procedures for detecting lipid rafts were applied before and after MβCD treatment (Figure 2), in which filipin was used to detect cholesterol (Figure 2A) [22] and FITC-conjugated cholera toxin B was used to label GM1 gangliosides expressed in lipid rafts (Figure 2B) [31].

The examples of Figure 2A show filipin staining in neurons (marked by arrow in right panel) as well as in surrounding satellite cells (arrow): the signal intensity was significantly (p = 0.03) stronger for KI than WT cultures and, in both WT and KI cultures, MβCD treatment (30 min) decreased, on average, the filipin signal intensity (p = 0.02). Nonetheless, since filipin labels cholesterol that is widely distributed throughout cells, a more specific signal was sought with CTX-B that also provided significantly (p < 0.001) stronger signal from KI rather than WT cells (Figure 2B). This signal was decreased after MβCD in both genotypes (Figure 2B). The effective disruption of lipid rafts by MβCD was confirmed by an altered distribution of P2X3 receptors as exemplified by western immunoblotting data (Figure 2C, left). In accordance with in situ ganglion results shown in Figure 1D, the P2X3 receptor signal was larger in raft fractions (R) versus non-raft membranes (NR) from KI cultures with respect to WT (Figure 2C, right). Furthermore, as a consequence of lipid disruption by MβCD pre-treatment, a significant redistribution of P2X3 receptor expression was observed in NR fractions of both WT and KI samples (Figure 2C, right). The P/Q channel blocker ω-agatoxin (300 nM; overnight) caused strong reduction in the lipid raft membrane fraction of KI cells without a significant change in the WT ones (Figure 2D, p = 0.02, n = 4), thus linking the Ca_V_2.1 gain-of-function to the larger lipid raft expression by KI membranes.

In summary, our data concur to indicate that KI neurons were enriched with lipid rafts where the major fraction of membrane P2X3 receptors was present.

### Lipid rafts dependent modulation of P2X3 receptor function

**Figure 3:**
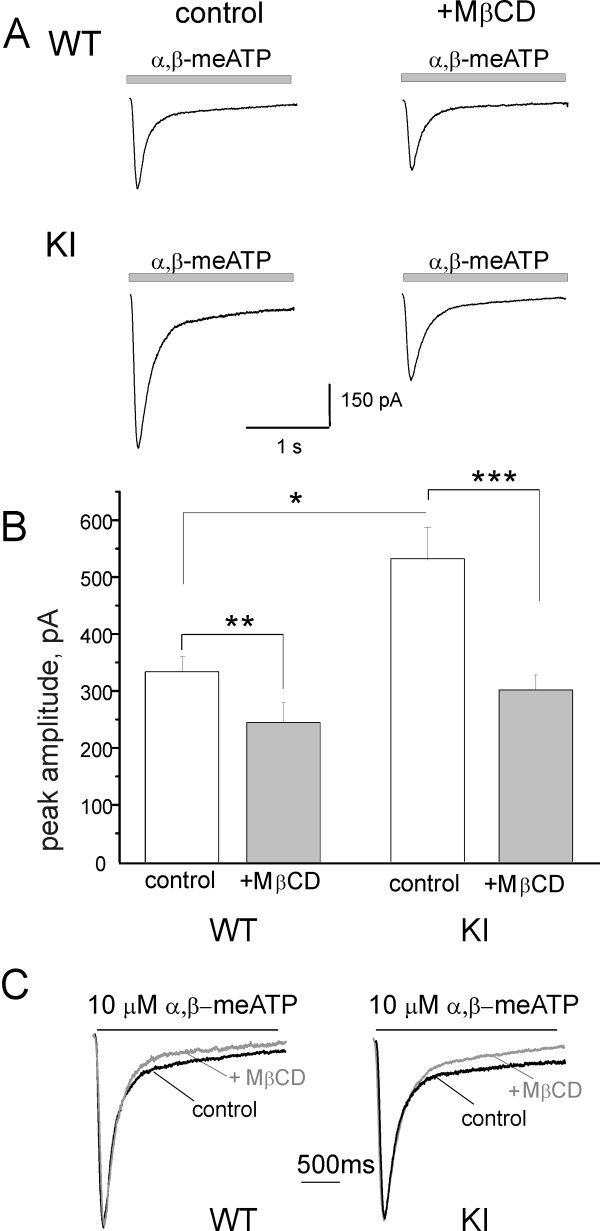
**Patch clamp recording from WT and KI cultured neurons**. A, Representative current traces evoked by α,β-meATP (10 μM, 2 s) from WT and KI neurons in control and after MβCD pre-treatment (30 min, 10 mM). B, Histograms quantify the current amplitude before or after MβCD pre-treatment for WT and KI neurons. Control: n = 33 for WT and KI. *: p = 0.006. **:p = 0.008; ***: p = 0.002. MβCD: n = 30 for WT and KI. C, P2X3 mediated current traces (average of 14-20 responses) in control (black) and after MβCD pre-treatment (gray), normalized and overlapped, from WT (left) and KI (right) neurons. Note no change in the current onset, and clear acceleration of its slow decay.

Patch-clamp recording allowed us to study whether the differential distribution of P2X3 receptors between WT and KI neurons might have a functional consequence. To this end, we compared the effect of MβCD pretreatment on WT and KI neurons whose P2X3 receptors were activated to produce large and fast decaying inward currents by a pulse of α,β-methylene ATP (α,β-meATP; 10 μM), a selective agonist of this receptor subtype [32], as exemplified in Figure 3A. On average, we first confirmed that the peak amplitude of KI currents was larger than the WT ones (520 ± 58 vs. 338 ± 26 pA, n = 33; p = 0.006). Although MβCD did not significantly change neuronal input resistance (420 ± 66 and 430 ± 71 MΩ before and after MβCD), it did, however, decrease the peak amplitude of α,β-meATP mediated currents (p = 0.008 for WT and p = 0.002 for KI) by about 30% for WT and 44% for KI cells to similar values (Figure 3A, B).

MβCD application led to changes in cell capacitance: from 32 ± 2 to 20 ± 2 pF for WT (p < 0.001), and from 29 ± 2 to 23 ± 2 for KI (p = 0.04). Thus, we also calculated the responses evoked by α,β-meATP (10 μM) in terms of current density rather than peak amplitude. Hence, control WT responses were, on average, 11.7 ± 1.1 pA/pF (n = 30), while KI responses were 18.1 ± 1.7 pA/pF (n = 30; p = 0.003). Following MβCD application, the corresponding values were 13.8 ± 1.6 pA/pF (n = 22) for WT and 14.5 ± 1.4 pA/pF for KI (n = 24; p = 0.74) for KI neurons. Hence, the significant difference in P2X3 receptor mediated responses between WT and KI neurons was lost after MβCD application.

**Figure 4:**
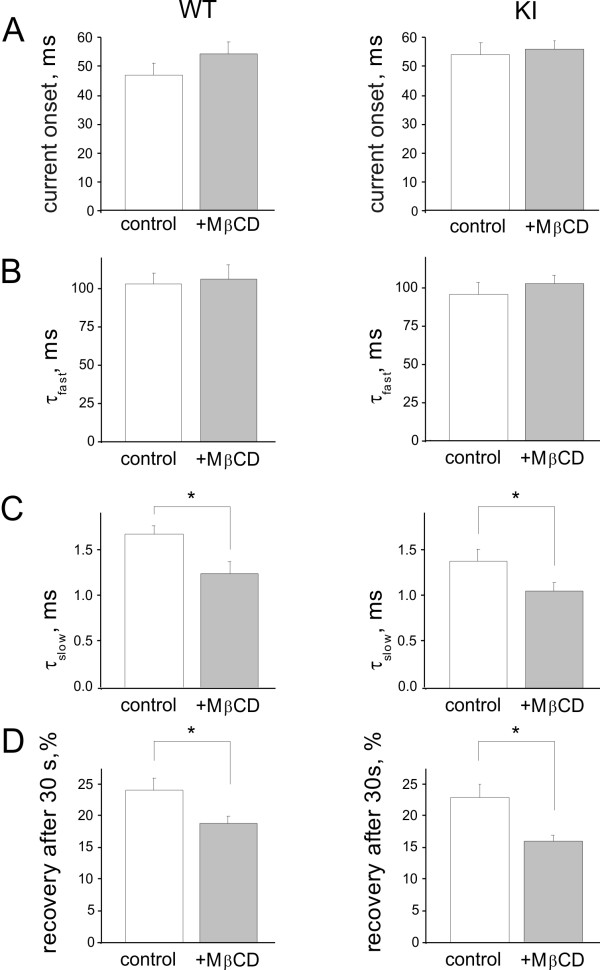
**Properties of P2X3 receptor mediated responses of WT and KI neurons**. Histograms quantify the different properties of P2X3 receptors in WT (left) or KI (right) trigeminal neurons in control and after MβCD treatments. Note that MβCD treatment accelerates the slow desensitization time constant and slows down the degree of recovery from desensitization in WT and KI neurons (tested at 30 s interval). Data refer to effects induced by 10 μM α,β-meATP. A, current onset expressed as 10-90% response rise time. Control: n = 20 for WT and n = 25 for KI. MβCD: n = 15, p > 0.05 for WT; n = 27, p > 0.05 for KI. B, desensitization onset (fast desensitization time constant, τ_fast_) Control: n = 18 for WT and n = 20 for KI. MβCD: n = 12, p > 0.05 for WT and n = 24, p > 0.05 for KI. C, retarded current decay (slow desensitization time constant, τ_slow_). Control: n = 18 for WT and n = 20 for KI. MβCD: n = 11, p < 0.01 for WT and n = 21, p = 0.04 for KI. D, recovery from desensitization, expressed as % of control response amplitude with 30 s paired pulse protocol of α,β-meATP application. Control: n = 12 for WT and n = 16 for KI. MβCD: n = 7, p = 0.03 for WT and n = 11, p < 0.01 for KI.

Figure 3C compares examples (on a faster time base) of scaled and superimposed currents recorded before and after MβCD application. For both WT and KI responses, there was a clear acceleration of the slow component of current decay. Figure 4 shows that neither the current onset (A) nor the fast current decay (τ_fast_, B) was changed by MβCD. However, the slow decay of the current (measured as τ_slow_; Figure 4C) was accelerated, likely reflecting changes in late phases of receptor desensitization [33]. This process can explain the delayed recovery from desensitization (Figure 4D; tested with the standard protocol of two paired pulses of α,β-meATP spaced 30 s apart; [27,33]). These data suggest that depletion of cholesterol by MβCD led to diminished P2X3 receptor current amplitude with accelerated desensitization and slower recovery from it in WT and KI neurons.

### Kinetic modeling of the P2X3 receptor changes following MβCD application

**Figure 5:**
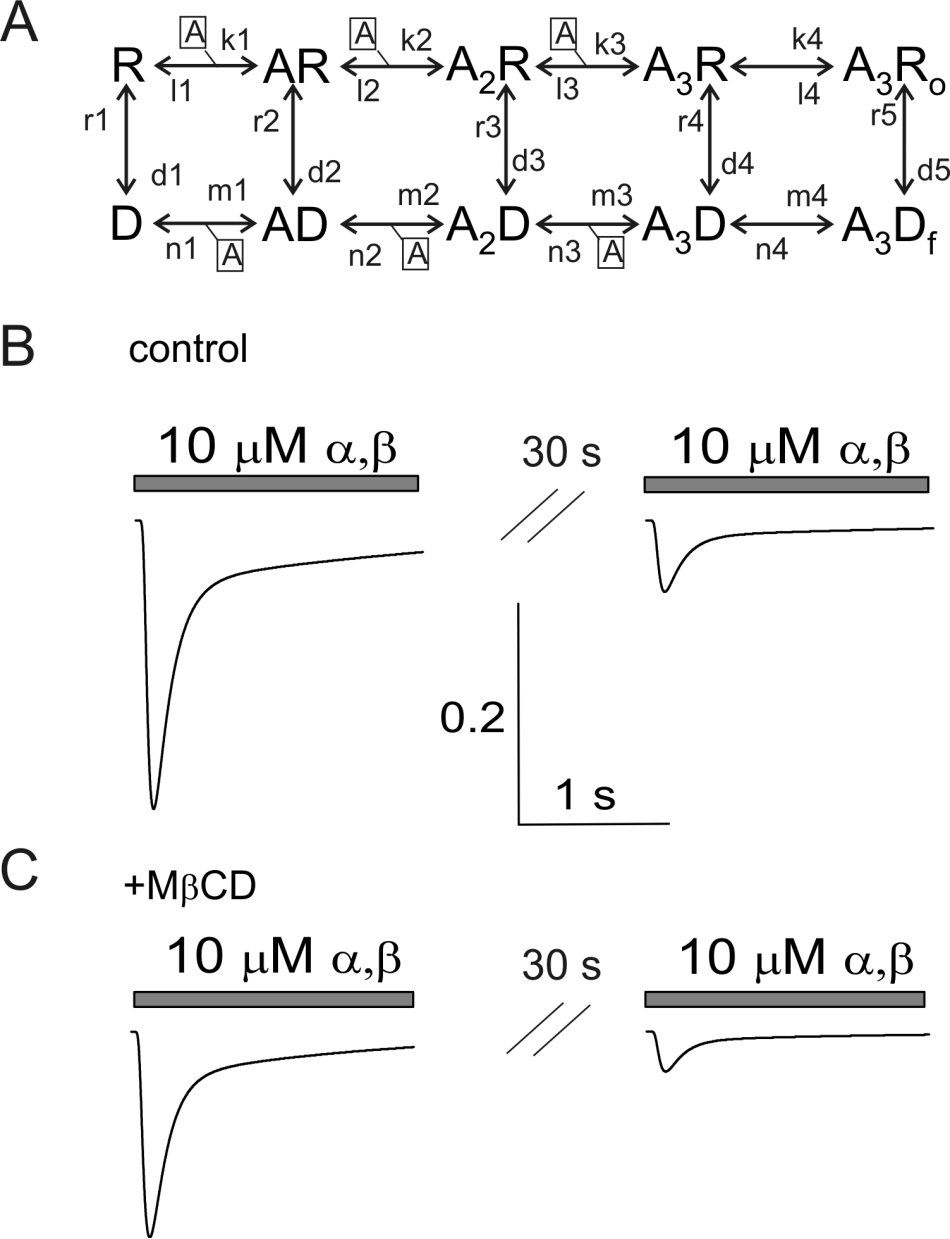
**Modeling changes in P2X3 receptor activity after MβCD**. A, Scheme of the cyclic model of P2X3 operation with reversible multi step process adapted from [33]. A, agonist (suffix values indicate the number of bound molecules); R, resting state; A_3_R_o_, fully-occupied open state; A_3_D_f_, rapidly desensitizing state; AR_n _and AD_n_, bound non activated and desensitized states, respectively, with corresponding number of bound agonist molecules. Forward rate constants for activation are indicated as k, while reverse rate constants are indicated by l. The process of desensitization is indicated by n or m constants depending on the reaction direction. Intermediate transitions from each one of the upper and lower branch states are possible with rate constants indicated as r or d. For the purpose of simulating the actual experimental conditions observed with rapid, short pulse of agonist application at high concentration, the scheme is envisaged to primarily follow the R, A_3_R_o_, A_3_D_f_, D pathway. Rate constant values are provided in Table 1 after adapting data from rat dorsal root ganglion neurons [33]. B, Simulated responses evoked by paired pulses of α,β-meATP (10 μM, 2 s; 30 s interval) in control condition. The amplitude of the second response recovers to 24% of the first one, in analogy with experimental data. C, Similar protocol simulated after MβCD treatment. Note that the current amplitude is diminished, and the current decay is faster, while recovery from desensitization is impaired. The vertical calibration bar refers to the fraction of active receptors.

We next explored the changes in the functional activity of the P2X3 receptors by using a cyclic kinetic model of receptor operation in which various phases of activation and desensitization are included (see Figure 5A; [33]). In the case of P2X3 receptors, kinetic modelling is advantageous to explore certain receptor operation steps which develop very rapidly and are readily obscured by intervening desensitization, making quite difficult to extract detailed information from single channel studies [33,34].

Hence, by optimization of the rate constants in this kinetic model (see Materials and Methods), we could adequately simulate α,β-meATP (10 μM) evoked currents in terms of experimental amplitude, current onset, desensitization time constants (τ_fast _and τ_slow_), and the degree of recovery from desensitization tested 30 s later (Figure 5B). The corresponding rate constants are provided in Table 1. It should be noted that these values are the rate constants optimized to simulate the present mouse trigeminal neuron data as the original model was derived from rat P2X3 receptors of dorsal ganglion neurons [33]. Once the actual currents observed with patch-clamping were closely replicated by the model, changes in discrete reaction steps were tested. Figure 5C shows how changes in the values of rate constants controlling the process of receptor activation and desensitization could closely replicate all the effects observed experimentally after MβCD without affecting parameters like the current onset or its fast desensitization (see Figure 4). In particular, reduced current amplitude was produced by decreasing the rate constant k4 (see Figure 5A and Table 1) to slow down gating of the active fully-bound receptor. This simple change, however, was associated with slower current decay (not shown) rather than the faster decline observed experimentally. Thus, to closely mimic the accelerated decay of the slow current component together with smaller recovery from desensitization, we increased the rate constant d5 of receptor transition from the open bound state A_3_R_o _to the rapidly desensitizing A_3_D_f _as well as the rate constant n4 from the A_3_D_f _state to the slower desensitizing bound state A_3_D.

**Table 1:** Values of rate constants of P2X3 receptor kinetic model in mouse trigeminal neurons

Rate Constant	Control	+ MβCD
k1	24000 (mM^-1^·s^-1^)	24000 (mM^-1^·s^-1^)
l1	15 (s^-1^)	15 (s^-1^)
k2	16000 (mM^-1^·s^-1^)	16000 (mM^-1^·s^-1^)
l2	30 (s^-1^)	30 (s^-1^)
k3	8000 (mM^-1^·s^-1^)	8000 (mM^-1^·s^-1^)
l3	45 (s^-1^)	45 (s^-1^)
k4	75 (s^-1^)	40 (s^-1^)
l4	10 (s^-1^)	10 (s^-1^)
m1	24000 (mM^-1^·s^-1^)	24000 (mM^-1^·s^-1^)
n1	0.01 (s^-1^)	0.019 (s^-1^)
m2	16000 (mM^-1^·s^-1^)	16000 (mM^-1^·s^-1^)
n2	0.02 (s^-1^)	0.038 (s^-1^)
m3	8000 (mM^-1^·s^-1^)	8000 (mM^-1^·s^-1^)
n3	0.03 (s^-1^)	0.057 (s^-1^)
m4	0.001 (s^-1^)	0.001 (s^-1^)
n4	0.44 (s^-1^)	0.68 (s^-1^)
d1	0.00001 (s^-1^)	0.00001 (s^-1^)
r1	10 (s^-1^)	10 (s^-1^)
d2	0.12 (s^-1^)	0.12 (s^-1^)
r2	0.00001 (s^-1^)	0.00001 (s^-1^)
d3	0.00001 (s^-1^)	0.00001 (s^-1^)
r3	0.00001 (s^-1^)	0.00001 (s^-1^)
d4	0.00001 (s^-1^)	0.00001 (s^-1^)
r4	0.00001 (s^-1^)	0.00001 (s^-1^)
d5	9.0 (s^-1^)	13.0 (s^-1^)
r5	1.1 (s^-1^)	1.1 (s^-1^)

## Discussion

The principal finding of this study is the novel demonstration that in KI mice P2X3 receptors of trigeminal neurons are preferentially localized to lipid rafts, a phenomenon associated with a molecular phenotype characterised by stronger responses to P2X3 receptor activation [27]. Because altering lipid rafts was translated into less efficient P2X3 receptor mediated responses, our data suggest that P2X3 receptor compartmentalization at membrane level contributed to the larger ATP signalling by KI neurons.

### A phenotype with gain-of-function of P2X3 receptors contained a larger membrane lipid raft domain

KI trigeminal neurons carrying the R192Q missense mutation in the α1 subunit of Ca_V_2.1 channels are known to express enhanced P2X3 receptor activity [27]: this phenomenon is attributed to the larger intracellular Ca^2+ ^levels mediated by hyperfunctional Ca_V_2.1 channels (P/Q type) and a consequent change in P2X3 receptor phosphorylation state without alteration in their total membrane expression. The present investigation expanded these findings by adding that KI neurons had significantly larger association of P2X3 receptors to the lipid raft compartment. This association might have important functional implications because lipid rafts can form concentrating platforms for individual receptors activated by ligand binding [35]. For instance, distinct intracellular signalling pathways have been proposed for P2X_7 _receptors when distributed to raft or non-raft domains [36]. Previous studies have indicated that Ca_V_2.1 channels are particularly clustered in lipid microdomains [37], thereby placing them in a strategic position to directly modulate lipid rafts and their associated P2X3 receptors. In support of this notion, the present report showed that there was a significant decrease in the membrane lipid raft fraction of KI samples after pretreatment with the Ca_V_2.1 channel blocker ω-agatoxin. It seems, therefore, likely that the gain of function of mutated Ca_V_2.1 channels led to upregulated lipid raft expression by KI cells. Nonetheless, the present report does not exclude the possibility that increased intracellular Ca^2+ ^due to the mutated channels might have also operated via intracellular pathways [20] to functionally modify P2X3 receptors resident in raft domains.

To investigate the impact of lipid rafts on P2X3 receptor activity, we tested the consequences of altering the composition of lipid rafts with MβCD. We detected, after such a treatment to trigeminal neurons, a small reduction in cholesterol (labelled with filipin that marks all cholesterol stores), and a more evident decrease in CTX-B positive domains (corresponding to membrane lipid rafts). These data, thus, suggested that lipid rafts were pivotal to determine stronger P2X3 receptor activity of KI neurons. To further test this hypothesis, we decided to decrease lipid rafts and their P2X3 content, and to find out if P2X3 receptor function of KI neurons reverted to the WT phenotype.

### Functional changes of P2X3 receptors evoked by altering lipid rafts

Electrophysiological studies showed similar impairment in the function of P2X3 both in WT and KI after MβCD treatment. Thus, the mere presence of P2X3 receptors in lipid rafts did not confer them special intrinsic properties, rather it favoured their assembly within a macromolecular structure that possibly contributed to the global cell responses constitutively larger in KI neurons [27]. It seems, therefore, likely that the more abundant lipid rafts of KI neuronal membranes simply created a more favourable environment to express P2X3 receptors and to maximize their activity. This notion accords with the suggestion that lipid rafts provide a spatial and temporal meeting point for signalling molecules, as they contain a number of proteins that might be transiently or permanently associated with ion channels [38,39]. Channel function can be modulated by lipid rafts in a number of different ways through direct lipid-protein interactions or by altering the biophysical properties of the lipid bilayer. For instance, in the field of purinergic receptors, phosphoinositides can modulate the function of P2X3 [40] and P2X4 receptors by interacting with their C-terminal [41]. In addition, lipid rafts may be the milieu to promote receptor trafficking and internalization [23]. Notwithstanding the need to clarify the molecular interactions between P2X3 receptors and lipid rafts with future studies, the present report provides evidence that the MβCD-induced alteration in lipid rafts was accompanied by changes in the current amplitude and desensitization of P2X3 receptors. Because cholesterol is an essential component of cell membranes, it was not surprising that MβCD could decrease the cell capacitance. Even when data were calculated in terms of current density to take this change into consideration, there was no significant difference between WT and KI responses following MβCD application. Thus, non-raft P2X3 receptors whether of WT or KI cells appeared to share similar effectiveness, leading to the suggestion that the milieu of the lipid raft domain likely conferred stronger activity to P2X3 receptors. As lipid rafts were more abundant in KI neurons, they, therefore, represented an important substrate to facilitate P2X3 receptor mediated responses. Recombinant P2X3 receptors expressed by HEK cells did not apparently change their function after MβCD [17]; the discrepancy from the present native receptors might be attributed to differences in the lipid raft compartments of HEK cells versus sensory ganglion neurons.

Non-specific changes in cell properties could not account for the stronger receptor desensitization because smaller size responses (due to the activity of fewer receptors) are normally associated with less desensitization [33,42]. Indeed, our kinetic model showed that simulating only impaired receptor activation was translated into slower response decay, unlike the faster current decay actually detected experimentally.

Previous studies of ligand-gated receptors have exploited kinetic modelling to unravel conformational states that cannot be resolved at the macroscopic level and to provide a detailed mechanism of channel activation [43]. In the case of P2X3 receptors with fast activation and desensitization, kinetic modelling contributes to the understanding of these mechanisms by revealing additional information that is not readily available from single-channel recording. Indeed, kinetic modelling has largely helped to clarify detailed operation, for example, of glutamate [44,45], P2X2 [46] or nicotinic receptors [47]. The cyclic scheme of P2X3 receptor operation has been subsequently refined by including an allosteric mechanism useful to account for the phenomenon of very slow desensitization evoked by ambient ATP [34]. Because the present report was focussed on rapid responses induced by pulse application of α,β-meATP, it was felt unnecessary introducing these additional steps. The use of this kinetic model remains, of course, a simplification of the receptor operational mode useful to supply a scheme compatible with the interpretation of the observed results. Reducing the rate constant controlling the isomerization of the fully-occupied receptor could reproduce the smaller amplitude without changing the response onset. Furthermore, faster transition from active to the desensitized receptor state replicated the accelerated current decay.

These observations closely resemble the behaviour described for GABA_A _receptors when their lipid microenvironment is perturbed with consequent loss of response amplitude and increased rate of desensitization [48]. Likewise, lipid microenvironment in form of "shell" or "annular" lipids has a strong effect on the structural and functional properties of the nicotinic receptors [49-51] as cholesterol is an absolute requirement for their stability, supramolecular organization and function [52-54].

## Conclusions

The present study reports the novel finding that KI neurons expressing a genetic mutation associated with familial hemiplegic migraine were enriched in their membrane microdomain of lipid rafts where P2X3 receptors were predominantly housed. The stronger association of such receptors with lipid rafts had the functional consequence of evoking larger responses mediated by these nociceptors that are important transducers of acute trigeminal pain.

## Materials and methods

### Genetic Model

Ca_V_2.1 α1 R192Q mutant KI and WT littermates were used for the experiments. Genotyping was routinely performed as previously described [25,27]. Homozygous R192Q KI and WT littermates used for this study share the same genetic background (~97% C57Bl6J background), as described in van den Maagdenberg et al [25], and originally backcrossed for five generations followed by further in-house backcrossing to preserve the original phenotype. All procedures were approved by the local ethical committee for animal experimentation in accordance with guidelines of the International Association for the Study of Pain. Animals were maintained in accordance with the Italian Animal Welfare Act and their use was approved by the Local Authority Veterinary Service.

### Cell culture preparation of TG neurons

Trigeminal ganglia primary cultures from KI and WT mice were prepared as previously described (P10-14), and used after 24 h from plating [29,55].

### Membrane protein extraction

For total membrane protein extraction, trigeminal ganglia were lysed in phosphate saline buffer (PBS) containing Triton X-100 1%, 100 mM NaF, 20 mM orthovanadate plus the protease inhibitors cocktail (Sigma, Milan, Italy), incubated on ice for 45 min and centrifuged for 20 min at 10,000 *g *at 4°C. Total ganglia membranes were subjected to ultracentrifugation at 100,000 *g *for 1 h (4°C) with a fixed-angle rotor [56,57]. Triton-insoluble membrane pellets were dissolved in sample buffer (100 mM Tris-HCl pH 6.8, 200 mM dithiothreitol, 4% SDS, 20% glycerol, 8 M urea) and separated on 8% polyacrylamide gel, and represented the raft fraction. The remaining supernatant (Triton-soluble) was defined as non raft fraction.

To ensure correct equal loading for neuronal cell content in different lysates, protein extracts were quantified with bicinchonic acid (Sigma) and normalized for the neuronal specific β-tubulin III. The amount of loaded proteins was in the 20-50 μg/mL range. Immunoprecipitation of P2X3 receptors from the two fractions (raft and non-raft) was performed as described earlier [57]. β-tubulin or β-actin were used for gel loading reference.

### Sucrose density gradient preparation

Sucrose density gradients were prepared as described previously [58]. Briefly, cells were lysed in TNE buffer (10 mM Tris HCl pH 7.4, 150 mM NaCl, 2 mM EDTA, 20 mM NaF, 20 mM orthovanadate) plus protease inhibitors (30 min on ice) followed by sonication; insoluble membrane proteins were purified by ultracentrifugation (100,000 *g *for 60 min at 4°C). Pellets were resuspended in 500 mM Na_2_CO_3_, and added to 25 mM 2-N-morpholino ethanesulfonic acid (MES buffer), 150 mM NaCl, at pH 6, with 90% sucrose and protease inhibitors to reach a final dilution of 45% sucrose. The lysate was transferred to a TLS55 ultracentrifuge tube with a swing-bucket rotor to separate lipid rafts [15], and layered sequentially with 90, 45 and 5% sucrose in MES buffer. Samples were centrifuged at 200,000 *g *for 16 h and 100 μl fractions were collected from the top. 1% CHAPS was added to each fraction (to allow correct migration in western blotting) that was diluted with TNE buffer (1:5) and kept for 45 min on ice. Proteins were concentrated by centrifugation at 13,000 *g *for 30 min at 4°C. Final pellets were resuspended in sample buffer (100 mM Tris-HCl pH 6.8, 200 mM dithiothreitol, 4% SDS, 20% glycerol, 8 M urea) and separated on 8% polyacrylamide gel.

### Western immunoblotting

Western immunoblotting was performed using the following antibodies: rabbit anti-P2X3 (1:300; Alomone, Jerusalem. Israel), mouse anti-flotillin 1 (1:250, BD Biosciences, Franklin Lakes, NJ, USA), mouse anti β-actin (specific HRP-conjugated; dilution 1:1000; Sigma) and mouse β-tubulin III (1:1000, Sigma).

As secondary antibodies, to avoid detection of immunoglobulin heavy chains in Western blot, previously-validated HRP-conjugated antibodies (Jackson ImmunoResearch, Suffolk, UK) were used [57]. Signals were detected with the enhanced chemiluminescence light system ECL (Amersham Biosciences, Piscataway, NJ). For quantification of intensities of the immunoreactive protein bands we used Scion Image software (NIH, Bethesda, USA) or the digital imaging system UVTEC (Cambridge, UK).

### Cholesterol and rafts labelling

Cholesterol distribution in cultured trigeminal neurons was analyzed using a cell based cholesterol assay kit (Cayman, Ann Arbor, MI) [59,60], based on filipin staining (5 μg/mL, 10-20 min), a compound that forms fluorescent complexes with unesterified cholesterol [22].

For detection of lipid rafts, paraformaldehyde fixed trigeminal neurons were washed with PBS and incubated for 30 min with blocking solution (5% FCS, 5% BSA, 0.1% Triton-X) followed by 3 times washout with PBS, and then incubated with FITC conjugated cholera toxin B subunit (3 μg/mL; Sigma) for 10 min and then washed thrice with PBS. Labelled cells were viewed with a Zeiss microscope and acquired with MetaView software (Molecular Devices, Downingtown, PA, USA) in non-saturation mode. To quantify data, basal threshold was arbitrarily set to zero, and grey values were then analyzed with MetaMorph software (Molecular Devices, Downingtown, PA, USA). Response intensity was evaluated for each cell from each region of interest (0.075 mm^2^) and expressed as arbitrary units (AU). An average of fifty cells was analyzed in each test; data are the mean of at least three independent experiments.

### Patch Clamp Recording

Currents were recorded from mouse trigeminal neurons in culture as previously described [27] under whole cell voltage clamp mode at a holding potential of -60 mV. Cells were continuously superfused with control solution containing (in mM): 152 NaCl, 5 KCl, 1 MgCl_2_, 2 CaCl_2_, 10 glucose, 10 HEPES; pH 7.4 adjusted with NaOH. Patch pipettes had resistance of 3-4 MΩ when filled with (in mM): 140 KCl, 2 MgCl_2_, 0.5 CaCl_2_, 2 ATP-Mg, 2 GTP-Li, 20 HEPES, 5 EGTA; pH 7.2 adjusted with KOH. Data were acquired and analyzed with the pCLAMP software Clampex 9.2 (Molecular Devices, Palo Alto, CA, USA).

### Drug application

The agonist α,β-meATP (Sigma) was applied for 2 s by rapid solution changer system (Perfusion Fast-Step System SF-77B, Warmer Instruments, Hamden, CT, USA). The cholesterol depleting agent methyl-β-cyclodextrin (MβCD; Sigma) was dissolved in water and then applied to the cells in culture at the concentration of 10 mM for 30 min in the cell incubator in accordance with previous reports [22,61]. Electrophysiological recordings started after 10 min of wash with control solution and continued for approximately 1 h. This protocol of MβCD application increased the holding current of patch-clamped neurons from -50 ± 9 to -85 ± 9 pA (n = 65 and 49; p = 0.01).

### Data Analysis

The currents were analyzed in terms of their peak amplitude and current onset (10-90% of the response rise-time). The decay of the current during agonist application due to receptor desensitization was fitted with a biexponential function using Origin 6.0 (Microcal, Northampton, MA, USA) which provided the desensitization time constants (τ_fast _and τ_slow_). Recovery from desensitization was assessed by a paired pulse protocol over 30 s intervals in accordance with previous reports [27,55].

All data are presented as mean ± standard error of the mean (S.E.M); n is number of cells. Statistical significance was evaluated with unpaired Student's t-test (for parametric data) or Mann-Whitney-Wilcoxon test (for nonparametric data), p ≤ 0.05 was considered significant.

### Kinetic modeling

Kinetics of the P2X3 receptor mediated currents was simulated using the cyclic Markov state model [33] shown in Figure 5A. It assumes the binding of the three molecules of the agonist to the receptor in the resting state R, the isomerisation of the receptor into the open state R_o_, followed by transition into the rapidly developing desensitized state D_f_, and the slower occurring desensitized-bound state A_3_D with slow agonist dissociation. Standard desensitization is inferred to develop from open bound channels A_3_R_o_.

In total, in the model there are 10 states and 26 rate constants. The original model used rate constants applied to membrane currents recorded from rat dorsal root ganglion neurons. To adapt this scheme to trigeminal mouse sensory neurons that possess distinct time-course of desensitization and recovery [29], manual optimization of the rate constants was performed, using a PC, with our in-house developed program [33] written in Delphi. Hence, the peak amplitude, onset and decay time constants and recovery of the α,β-meATP induced P2X3 receptor mediated currents in control conditions and after cholesterol depletion with MβCD were obtained with the rate constant values indicated in Table 1 to reproduce the experimentally-obtained records. As we observed a comparable experimental change in WT and KI responses after MβCD, only simulation of the WT currents is shown.

## List of abbreviations used

α,β-meATP: α,β-methylATP; CTX-B: cholera toxin B; FHM-1: familial hemiplegic migraine type 1; τ_fast: _time constant of fast current decay; GPI: glycosylphosphatidylinositol; MβCD: knock-in, KI; methyl β-cyclodextrin; PBS: phosphate buffered saline; τ_slow: _time constant of slow decay of the current; TG: trigeminal ganglia; WT: wild type.

## Competing interests

The authors declare that they have no competing interests.

## Authors' contributions

All authors read and approved the final manuscript. MS and AG provided equal contribution to this study. EF, AN and AG design of experiments; MS, functional studies and modeling; AG, molecular biology experiments; AMJMVDM, supply of genetic model and data discussion; AN, MS, AG, AMJMVDM and EF, joint contribution to MS writing.
